# Psychometric evaluation of an interview-administered version of the WHOQOL-BREF questionnaire for use in a cross-sectional study of a rural district in Bangladesh: an application of Rasch analysis

**DOI:** 10.1186/s12913-019-4026-0

**Published:** 2019-04-05

**Authors:** Mohammed Nazim Uddin, Fakir M Amirul Islam

**Affiliations:** 1 Department of Statistics, Data Science and Epidemiology; Faculty of Health, Arts and Design; Swinburne University of Technology, Hawthorn, VIC 3122 Australia; 2 Organisation for Rural Community Development (ORCD), Dariapur, Narail, Bangladesh

**Keywords:** WHOQOL-BREF, Quality of life, Rasch analysis, Validation, Rural Bangladesh, Classical test theory

## Abstract

**Background:**

This study aimed to validate the psychometric properties of the World Health Organization Quality of Life Instrument, Short Form (WHOQOL-BREF) questionnaire for use in a rural district of Bangladesh.

**Methods:**

This cross-sectional study recruited a multi-stage cluster random sample of 2425 participants from the rural district Narail of Bangladesh in May–July 2017. Rasch analysis was carried out using the sampled participants, as well as multiple validation random sub-samples of 300 participants, to validate four domains of the WHOQOL-BREF questionnaire: physical, psychological, social and environmental.

**Results:**

The original WHOQOL-BREF appeared to be a poor fit for both sampled and sub-sampled group of participants in Narail district in all underlying domains: physical, psychological, social and environmental. Two items (*sleep* and *work capacity*) from the physical domain, two items (*personal belief* and *negative feelings*) from the psychological domain and three items (*home environment*, *health care* and *transport*) from the environment domain were excluded for goodness of fit of the Rasch model. The social domain exhibited reasonably reliable fitness while fulfilling all the assumptions of the Rasch model. A modified version of the WHOQOL-BREF questionnaire using five-items for the physical (${\upchi}_{(20)}^2$ = 36.47, *p* = 0.013, Person Separation Index (PSI) = 0.773), four-items for the psychological (${\upchi}_{(16)}^2$ = 28.30, *p* = 0.029, PSI = 0.708) and five-items for the environmental (${\upchi}_{(20)}^2$ = 36.97, *p* = 0.011, PSI = 0.804) domain was applied, which showed adequate internal consistency, reliability, unidimensionality, and similar functioning for different age-sex distributions.

**Conclusions:**

The modified WHOQOL-BREF questionnaire translated into Bengali language appeared to be a valid tool for measuring quality of life in a typical rural district in Bangladesh. Despite some limitations of the modified WHOQOL-BREF questionnaire, further application of Rasch analysis using this version or an improved one in other representative rural areas of Bangladesh is recommended to assess the external validity of the outcomes of this study and to determine the efficacy of this tool to measure the quality of life at the national rural level.

**Electronic supplementary material:**

The online version of this article (10.1186/s12913-019-4026-0) contains supplementary material, which is available to authorized users.

## Background

In recent years, beyond conventional health measures, for example, mortality and morbidity, there has been an increasing focus on measuring quality of life (QOL) as an important outcome in clinical settings along with evaluations of the effects of various interventions on QOL, such as the effect of medicine [1, 2]. The World Health Organisation (WHO) defines QOL as “individuals’ perception of their position in life in the context of the culture in which they live and the value systems they have about their goals, expectations, standards and concerns” ([3], p., 1403).

The quality of life (QOL) is influenced by a range of factors that include physical wellbeing, mental state, psychological state, social connections, individual convictions and connections as salient features of the environment [4]. Moreover, social tension and difficulties can have a significant impact on health, affecting overall QOL [5]. Despite this, the majority of the examinations on QOL have concentrated on the effect of chronic diseases, for example, malignant growth, stroke, diabetes and HIV/AIDS [6–9], which have been assessed using different QOL measurement tools. Over the last two decades, various tools have been created to quantify QOL [10], but most of these are designed to measure QOL with respect to specific diseases [11–13], with a few exceptions [14–16]. As an exception, an American psychologist, John Flanagan, first developed the QOL scale [17, 18]; which provides a more generalised definition of QOL that can be used to assess QOL in an everyday context. The WHO Quality of Life (WHOQOL) 100 item tool was developed using cross-cultural, multinational studies on the concept of QOL [19]. However, in addition to broader difficulties associated with obtaining information about QOL from respondents, WHOQOL-100 may be too lengthy, incomprehensible and inconvenient for practical use. The WHOQOL-BREF is a shorter form of the WHOQOL-100 questionnaire; it is a 26-item instrument with items rated on a five-point Likert-type scale. This questionnaire has been used for extensive population studies [20]. However, the WHOQOL-BREF has not been applied in rural settings in any developing countries, including Bangladesh.

A Bengali version of the WHOQOL-BREF was developed in 2005 in a study of adolescents and adults residing in the capital city of Dhaka, Bangladesh, and its validity was assessed using the Classical Test Theory (CTT) [21, 22]. The tool was also applied in some other areas in Bangladesh [23, 24]. However, in the CTT approach, items and persons’ latent traits are measured separately, where the true scores are typically obtained by summing responses across items. It is assumed that items with similar underlying concepts are valued equally and the score dissimilarity between two nearby response scales is uniform. However, uniformity does not hold in most situations [25]. As a result, the CTT cannot be likened in an item-person continuum [26, 27]. In addition, CTT procedures treat raw scores and the responses of the items as interval data. These limitations can be solved rationally using Item Response Theory (IRT) modelling (Rasch Analysis) even though IRT also makes several assumptions, such as unidimensionality, invariability and local independence.

The primary limitations of Rasch models are related to their complicated mathematical equations that are hard for clinicians to understand [28]. However, Rasch models produce some useful statistics; reliability and separation indices, differential item functioning (DIF) or item bias and measurement invariance. These can be calculated directly from the IRT model [26, 27]. Moreover, Rasch analysis provides further advantages: each item can be individually analysed to regulate any redundancy, which may not be detected by CTT; item difficulty can be estimated; and an ordinal-to-interval modification table can be created to help clinicians to utilize the scale as a means to better comprehend the participant scores in accordance with Rasch algorithms [29, 30]. Previously, psychometric evaluations of the WHOQOL-BREF mainly used CTT methods. However, Rasch models are advantageous as they can detect items that are out-of-concept or redundant and can precisely measure the latent QOL of participants in rural areas using an ordinal-to-interval conversion table for a transformation of ordinal scores. Therefore, Rasch analysis is becoming an increasingly popular modern statistical method [31]. Moreover, the previously published studies are characterised by several limitations, such as small sample sizes and restricted samples, for example, samples involving only adolescent participants, only participants from slum areas, only participants with specific diseases or the study was conducted in an arsenic-affected area [21–24]. Therefore, generalisations of the results of studies suffer from external validity. Since the WHOQOL-BREF tool has not previously undergone a rigorous psychometric analysis in rural Bangladesh, the current research elaborates and takes advantage of the use of the Rasch model to fulfil this goal.

The purpose of this investigation was to apply Rasch analysis to the established four domains of the WHOQOL-BREF in order to conduct a detailed assessment of response format, item fit, dimensionality and targeting. Secondly, Rasch analysis was applied to assess the applicability of using all items within the four domains of the WHOQOL-BREF in the rural setting of Bangladesh.

## Methods

### Study population

Bangladesh is a country of 163 million people divided into 64 districts [32]. Two thousand four hundred and twenty-five adult Participants aged 18–90 years were recruited from the Narail district, which is located approximately 200 km south-west of Dhaka, the capital city of Bangladesh, between May and July of 2017. The study area, including its geographic location and population density, has been described in detail elsewhere [33].

### Sample size and statistical power

In most statistical cases, the *p*-value linked with the goodness of fit chi-square test is affected by the sample size. A small sample size can produce an unstable result, and this might jeopardise the generalisation of the findings. However, with a very large sample size, a small deviation from the Rasch model results in a significantly large chi-square value. A sample size of 250–500 participants allows precise estimates of the item and person location to be obtained [34–36]. A sample of approximately 300 is most suitable for Rasch analysis because large sample sizes can result in type I error whereby an item is falsely rejected as not fitting the Rasch model [34]. A sample size of 300 is considered to be large enough to provide 99% confidence that the estimated item difficulty is within ± ½ logit of its stable value [35]. Analysis was undertaken five times with five different random sub-samples, each of which was comprised of 300 participants that were randomly selected from the total sample of 2425 participants, to test the robustness of the scale. A separate analysis for each domain of the WHOQOL-BREF (physical, psychological, social and environmental) was performed. Since the first two items on the WHOQOL-BREF are not part of any domain, these were not examined further.

### Sampling frame

There are 8 divisions and 64 districts in Bangladesh, each district further subdivided into Upazila (sub-districts). The area within each sub-district, is located in the metropolitan areas (which are further divided into several wards, where each ward consists of multiple blocks locally known as ‘mahallas’), and in the rural areas (which are further divided into several unions, where each union is composed of various villages) [37]. A multi-stage cluster random sampling technique was used for this study. Three rural areas out of 13 unions and one urban/semi urban area out of nine wards of Narail Upazila were randomly selected at stage 1. Two to three villages or mahallas from each selected union or ward were randomly selected at the second stage, totalling 12 locations of data collection where 240–260 adults aged 18 to 90 years were interviewed from each location. The recruitment strategy and quality assurance in data collection were described in detail previously [33].

### WHOQOL-BREF questionnaire

The WHOQOL tools (WHOQOL-100 and WHOQOL-BREF) were developed using cross-cultural, multinational studies on the concept of QOL, across 15 countries and 30 centers globally [19]. The WHOQOL-BREF contains 26 items: 24 items of the WHOQOL-BREF were categorized to four domains (physical (7 items), psychological (6 items), social (3 items) and environmental (8 items) with two items not considered where one item measures overall QOL (item 1) and another item gauges the level of satisfaction with health (item 2). The chief investigator (AI) contacted the original developers of the WHOQOL-BREF team [38] to seek permission to use the Bengali version of the WHOQOL-BREF for research purposes in Bangladesh. The WHOQOL-BREF questionnaire was designed to collect data via two different methods: self-administered and interviewer-assisted/interviewer-administered. For the current study, data were collected using the interviewer-administered method and the scoring was performed according to the WHOQOL-BREF development guidelines [19]. Study participants were asked to indicate how satisfied they felt in each aspect of the domains during last 4 weeks using a Likert’s type scale ranging from 1 to 5 where 1 designates ‘very dissatisfied’ and 5 means ‘very satisfied’. Scores of each item were initially analysed using SPSS and further converted into RUMM2030 for validation purposes. Since trained interviewers performed the interviews and collected the data, the missing data was not significant, except for item 21 (*sexual activity*). Rasch assumptions for each domain were checked along with identification of opportunities to improve the domains. Table 1 refers to individual items in the WHOQOL-BREF questionnaire, including domain names and item numbers (in brackets).

**Table 1:** Individual items in the WHOQOL-BREF questionnaire, including domain names and item numbers (in brackets)

Overall quality of life (1) Satisfaction of health (2)			
Physical domain: Seven items Pain (3) Dependence of medical aids (4) Energy (10) Mobility (15) Sleep and rest (16) Activities of daily living (17) Work capacity (18)	Psychological domain: Six items Positive feeling (5) Personal belief (6) Concentration (7) Bodily image (11) Self-esteem (19) Negative feeling (26)	Social domain: Three items Personal relationship (20) Sexual activity (21) Social support (22)	Environmental domain: Eight items Security (8) Physical environment (9) Financial support (12) Accessibility of information (13) Leisure activity (14) Home environment (23) Health care (24) Transport (25)

### Outcome measure and covariates

The main outcome measure was the validity of the WHOQOL-BREF questionnaire using Rasch analysis. The socio-demographic variables of age, categorised as either adult (18 to 59 years) or older adult (60 to 90 years), in Bangladesh, population of age 60 years or older are typically classified as ‘elderly’ [39], and gender (male or female) were considered as DIF factors. Demographic details were collected for age, gender and level of education. Level of education was categorised as no schooling, primary school level of education (grade 1 to 5), secondary school level of education (grade 6 to 10), secondary school certificate (SSC) or higher secondary certificate (HSC) and at least a bachelor’s degree.

### Classical test theory (CTT)

This section describes the use and limitations of Classical Test Theory (CTT) for validating WHOQOL-BREF questionnaire and the rationale of preference of Rasch Model over CTT for this study. In terms of modern psychometric methods, the main alternatives to CTT are latent trait theories such as Item Response Theory (IRT) and Rasch analysis. The Rasch model is based on the relationship (probability) between the characteristics (difficulties) of the item and the individual’s ability. An assumption of scales developed through latent trait theories is unidimensionality (that is, that a single trait underlies all the items in the model). Similar to IRT, CTT is another fundamental measurement theory that researchers employ to construct measures of latent traits [40]; while IRT and CTT are similar, there are important differences between the two measurement systems. A more in-depth explanation of CTT [41–43] and IRT [44–47] can be found elsewhere. To date, the WHOQOL-BREF has primarily been validated using CTT, in which items and the person latent trait being measured are considered separately; therefore, they cannot be meaningfully and systematically compared [26, 27]. The limitations of CTT can be circumvented rationally using Rasch modelling [44, 45, 48–51].

### The Rasch model

The Rasch model was named after Danish mathematician Georg Rasch [52]. The model shows what should be expected in responses to items if measurement (at the metric level) is to be achieved. Two versions of the Rasch model are available: dichotomous [52] and polytomous [53]. The polytomous version of the Rasch model was used in this study.

The Rasch analysis employed in this study was conducted using the RUMM 2030 package [54]. The purpose of Rasch analysis was to maximise the homogeneity of the trait and to allow a more significant reduction of redundancy at no sacrifice to measurement information by decreasing item and scoring levels to yield a more valid and straightforward measure. The Rasch model makes some assumptions that need to be evaluated to ensure that an instrument has Rasch properties. The most commonly assessed Rasch assumptions are a) unidimensionality, b) local independence and c) invariability. According to the Rasch model, Chi-square item-trait interaction statistics define the overall fit of the model to the scale [55]. A non-significant chi-square *p*-value indicates that the hierarchical ordering of the items is consistent across all levels of the underlying trait. A Bonferroni adjustment of the level of significance value [56] is typically used to assess statistical significance. Item-person interaction statistics are presented as z-statistics with a mean of zero and a standard deviation (SD) of 1 (indicating perfect fit within the model). Individual item-fit statistics include the residuals (acceptable within the range ± 2.5) and a non-significant chi-square value [57].

The “threshold” parameter is represented by two response categories where either response is equally probable. In the case of polytomous items, it is essential to check whether the thresholds are ordered or not. Disordered thresholds indicate that the respondents are not able to discriminate between the response options. Disordered thresholds frequently result in item misfit and can be corrected by collapsing adjacent categories [58].

“Unidimensionality” is a basic Rasch model assumption. Unidimensionality implies that the scale measures only one construct, allowing the items to be summed together to form a scale with only one dimension [59]. A strict test of unidimensionality has been proposed by Smith [60]. For a scale to be unidimensional, less than 5% of the t-tests should be significant, or the lower bound of the binomial confidence interval should overlap 5% [61].

Item local independence means that, once the variance explained by the Rasch factor is removed, the items do not show further associations. The person-item residuals correlation matrix examines this, and it is expected that there is no relationship higher than 0.30. Christensen et al. [62] propose 0.2 is a reasonably stable value however, the consensus within the discipline is that 0.3 is a more appropriate value [63].

In Rasch measurement theory, the scale should also work in the same way irrespective of which group (e.g., gender or age) is being assessed [64, 65]. Differential item functioning (DIF) occurs when two sample groups with the same level of the construct measured respond to an item in a different way. In this study, sets of item difficulties were compared between genders (males vs. females) and between two age groups (adults 18–59 years vs. older adults 60–90 years). DIF is present when significant differences are obtained in the analysis of variance (ANOVA) with Bonferroni correction.

Scale targeting compares the distribution of person locations with item difficulties in the same scale, and is centred in zero logits [66]. In case of polytomous items, the thresholds distribution is taken. It is expected that the items or threshold locations cover the entire range of participants across the construct being measured. On the other hand, for a well-targeted measure (not too easy or not too hard for the participants) the mean (M) ± standard deviation (SD) of the person location should be 0 ± 1 logits. A negative mean value implies that the sample was located at a lower level of the construct than the average of the scale. Item difficulties should be adequately spread throughout the measure, and the covered range should not be too narrow. For most cases, a range − 5 to + 5 logits is considered sufficient. The distribution of item difficulties allows us to identify regions along the latent continuum that may be lacking items for reliable assessment.

Rasch analysis provides an indicator of reliability. In RUMM 2030, this is provided by the Person Separating Index (PSI) [67]. The PSI is equivalent to Cronbach’s alpha (CA), though it uses person estimates in logit instead of raw scores. Similar to CA, a value close to 1 indicates high internal consistency and a value less than 0.7 indicates model misfit [68].

## Results

Table 2 shows the summary statistics for both the validation sample and the complete data set, by gender. The mean (SD, range) age of the participants in the complete data set was 52.0 years (17 years,18–90 years). The demographic makeup of the total sample was 48.5% male and 51.5% female. Of the total participants, 27.6% did not have any formal education, 39% completed primary school and only 4% completed a bachelor’s degree or above. In the validation sample, which comprised of 300 participants, 50% were male and 50% were adults aged 18–59 years and the remaining 50% were older aduts aged 60–90 years.

**Table 2:** Demographic characteristics of participants by gender

Characteristic	Total sample *N* = 2425			Validation sample *N* = 300		
	Total (*N* = 2425) n (%)	Male (*N =* 1176) n (%)	Female (*N* = 1249) n (%)	Total (*N* = 300) n (%)	Male (*N* = 150) n (%)	Female (*N* = 150) n (%)
Adults (18–59 years)	1278 (52.7)	603 (51.3)	675 (54.0)	150 (50.0)	75 (50.0)	75 (50.0)
Older adults (60–90 years)	1147 (47.3)	573 (48.7)	574 (46.0)	150 (50.0)	75 (50.0)	75 (50.0)
Education						
No education	671 (27.7)	289 (24.6)	382 (30.6)	78 (26.0)	38 (25.3)	40 (26.7)
Primary education (1–5 years)	946 (39.0)	447(38.0))	499 (40.0)	119 (39.7)	53 (35.3)	66 (44.0)
Secondary education (6–9 years)	327 (13.5)	146 (12.4)	181 (14.5)	34 (11.3)	17 (11.3)	17 (11.3)
SSC^a^ or HSC^b^ Pass (10–12 years)	385 (15.9)	224 (19.0)	161 (12.9)	54 (18.0)	30 (20.0)	24 (16.0)
Degree or equivalent (13–16 years)	96 (4.0)	70 (6.0)	26 (2.1)	15 (5.0)	12 (8.0)	3 (2.0)

^a^SSC = Secondary School Certificate

^b^HSC = Higher Secondary Certificate

**Table 3:** Performance of the Rasch analysis of the WHOQOL-BREF domains (sample size, *n* = 2425)

WHOQOL-BREF scales	Original instrument					Summary of overall model fit statistics of each domain (original instrument)	
	Location	S. E.	Residual	χ^2^	*p-*value		
Overall							
Overall QOL (1)	0.07	0.04	−2.08	74.36	0.000		
General Health (2)	−0.07	0.04	−3.29	58.37	0.000		
Physical domain							
Pain (3)	−1.36	0.03	−1.57	101.14	0.000	Person separation index	0.825
Dependence on medical aids (4)	−1.35	0.04	3.95	96.71	0.000	Coefficient alpha	0.848
Energy (10)	1.12	0.03	7.49	137.15	0.000	Chi-square (Degrees of freedom)	765.18 (28)
Mobility (15)	0.22	0.03	−4.24	89.73	0.000	*p-*value	0.0000
Sleep and rest (16)	−0.39	0.03	2.93	111.44	0.000	Items fit residual (mean (SD))	−1.09 (6.12)
Activities of daily living (17)	0.97	0.04	−8.74	134.11	0.000	Persons fit residual (mean(SD)	−0.5 (1.19)
Work capacity (18)	0.79	0.04	−7.49	94.89	0.000		
Psychological domain							
Positive feeling (5)	0.15	0.03	−7.31	165.86	0.000	Person separation index	0.751
Personal belief (6)	0.64	0.03	−5.11	207.74	0.000	Coefficient alpha	0.763
Concentration (7)	−0.37	0.03	−7.31	117.79	0.000	Chi-square (Degrees of freedom)	843.59 (24)
Bodily image (11)	−0.33	0.03	3.19	94.61	0.000	*p-* value	0.0000
Self-esteem (19)	−0.89	0.03	5.83	183.12	0.000	Items fit residual (mean (SD))	−0.88 (6.36)
Negative feeling (26)	0.80	0.03	5.45	74.47	0.000	Persons fit residual (mean (SD))	−0.32 (1)
Social domain							
Personal relationship (20)	−0.41	0.03	−4.51	126.29	0.000	Person separation index	0.549
Sexual activity (21)	−0.14	0.03	−1.08	41.28	0.000	Coefficient alpha	^a^
Social support (22)	0.55	0.03	0.65	75.64	0.000	Chi-square (Degrees of freedom)	243.2 (12)
						*p*- value	0.0000
						Items fit residual (mean (SD))	−1.65 (2.63)
						Persons fit residual (mean (SD))	−0.37 (0.7)
Environmental domain							
Security (8)	−0.35	0.03	−2.46	53.73	0.000	Person separation index	0.716
Physical environment (9)	0.30	0.03	−0.7	28.62	0.000	Coefficient alpha	0.711
Financial support (12)	0.65	0.03	−10.14	211.65	0.000	Chi-square (Degrees of freedom)	859.57 (32)
Accessibility of information (13)	−0.06	0.03	−6.72	125.97	0.000	*p-*value	0.0000
Leisure activity (14)	−0.16	0.02	−0.71	17.56	0.001	Items fit residual (mean (SD))	−0.35 (6.25)
Home environment (23)	− 0.49	0.03	4.26	79.78	0.000	Persons fit residual (mean (SD))	−0.26 (1.05)
Health care (24)	0.15	0.03	4.66	70.61	0.000		
Transport (25)	−0.04	0.03	8.94	271.66	0.000		

^a^Coefficient of Alpha cannot be calculated on missing data

**Table 4:** Performance of the preliminary Rasch analysis of the WHOQOL-BREF domains (sub-sample (for validation) size, *n* = 300)

WHOQOL-BREF scales	Original instrument					Summary of overall model fit statistics of each domain (original instrument)	
	Location	S. E.	Residual	χ^2^	*p-*value		
Overall							
Overall QOL	−0.04	0.12	−1.29	2.5	0.645		
General Health	0.04	0.12	− 1.3	4.41	0.353		
Physical domain							
Pain (3)	−1.40	0.10	−0.23	15.22	0.004	Person separation index	0.834
Dependence on medical aids (4)	−1.98	0.10	1.91	18.41	0.001	Coefficient alpha	0.852
Energy (10)	1.24	0.09	1.91	8.50	0.075	Chi-square (Degrees of freedom)	107.18 (28)
Mobility (15)	0.19	0.09	−1.43	16.49	0.002	*p-*value	0.0000
Sleep and rest (16)	−0.28	0.09	1.80	24.03	0.007	Items fit residual (mean (SD))	−0.34 (2.39)
Activities of daily living (17)	1.63	0.10	−2.16	14.45	0.006	Persons fit residual (mean (SD))	−0.48 (1.17)
Work capacity (18)	0.61	0.10	−4.20	20.08	0.000		
Psychological domain							
Positive feeling (5)	0.23	0.09	−2.41	25.26	0.000	Person separation index	0.767
Personal belief (6)	0.55	0.08	−0.97	34.83	0.000	Coefficient alpha	0.776
Concentration (7)	−0.69	0.09	−2.31	13.77	0.008	Chi-square (Degrees of freedom)	130.64 (24)
Bodily image (11)	0.20	0.10	1.56	18.82	0.001	*p-*value	0.0000
Self-esteem (19)	−0.52	0.10	1.70	18.84	0.001	Items fit residual (mean (SD))	−0.09 (2.04)
Negative feeling (26)	0.22	0.08	3.87	19.13	0.001	Persons fit residual (mean (SD))	−0.22 (0.90)
Social domain							
Personal relationship (20)	−0.75	0.09	−1.85	15.36	0.004	Person separation index	0.605
Sexual activity (21)	−0.55	0.09	−0.61	9.96	0.041	Coefficient alpha	^a^
Social support (22)	1.31	0.09	0.52	21.90	0.000	Chi-square (Degrees of freedom)	47.21 (12)
						*p-*value	0.0000
						Items fit residual (mean (SD))	−0.64 (1.18)
						Persons fit residual (mean (SD))	−0.39 (0.71)
Environmental domain							
Security (8)	−0.66	0.08	−1.49	13.80	0.008	Person separation index	0.740
Physical environment (9)	−0.05	0.08	−0.86	6.94	0.139	Coefficient alpha	0.739
Financial support (12)	0.80	0.07	−3.99	34.08	0.000	Chi-square (Degrees of freedom)	173.69 (32)
Accessibility of information (13)	1.01	0.07	−2.54	20.62	0.000	*p-*value	0.0000
Leisure activity (14)	−0.19	0.07	−0.28	8.64	0.071	Items fit residual (mean (SD))	−0.12 (2.73)
Home environment (23)	−0.79	0.08	2.14	10.50	0.033	Persons fit residual (mean (SD))	−0.24 (1.07)
Health care (24)	0.53	0.09	1.62	22.32	0.000		
Transport (25)	−0.65	0.09	4.42	56.80	0.000		

^a^Coefficient of Alpha cannot be calculated on missing data

**Table 5:** Performance of the WHOQOL-BREF domains after Rasch model adjustment (sub-sample (for validation) size, *n* = 300)

WHOQOL-BREF Scale	Modified instrument					Summary of overall model fit statistics for each domain (modified instrument)	
	Location	S. E.	Residual	χ^2^	*p-*value		
Overall							
Overall QOL	−0.04	0.12	−1.29	2.50	0.645		
General Health	0.04	0.12	−1.30	4.41	0.353		
Physical domain							
Pain (3)	−1.41	0.09	−0.85	5.39	0.250	Person separation index	0.773
Dependence on medical aids (4)	−1.99	0.10	0.87	8.15	0.086	Coefficient alpha	0.790
Energy (10)	1.36	0.09	0.95	5.45	0.244	Chi-square (Degrees of freedom)	36.47 (20)
Mobility (15)	0.27	0.09	−1.35	10.64	0.031	*p-*value	0.0135
Sleep and rest (16)^a^						Items fit residual (mean (SD))	−0.31 (1.12)
Activities of daily living (17)	1.76	0.10	−1.15	6.84	0.145	Persons fit residual (mean (SD))	−0.52 (1.09)
Work capacity (18) ^a^							
Psychological domain							
Positive feeling (5)	0.46	0.10	−0.07	5.23	0.265	Person separation index	0.708
Personal belief (6) ^a^						Coefficient alpha	0.745
Concentration (7)	−0.46	0.09	−1.77	8.15	0.086	Chi-square (Degrees of freedom)	28.30 (16)
Bodily image (11)	0.36	0.10	0.72	9.89	0.042	*p-*value	0.029
Self-esteem (19)	−0.35	0.11	0.77	5.03	0.284	Items fit residual (mean (SD))	−0.09 (1.18)
Negative feeling (26) ^a^						Persons fit residual (mean (SD))	−0.30 (0.86)
Social domain							
Personal relationship (20)	−0.77	0.10	−1.59	14.05	0.007	Person separation index	0.635
Sexual activity (21)	−0.55	0.09	−0.84	10.08	0.039	Coefficient alpha	0.669
Social support (22)	1.32	0.09	0.71	18.96	0.001	Chi-square (Degrees of freedom)	43.09 (12)
						*p-*value	0.000
						Items fit residual (mean (SD))	−0.57 (1.18)
						Persons fit residual (mean (SD))	−0.38 (0.72)
Environmental domain							
Security (8)	−0.88	0.09	−0.05	12.24	0.016	Person separation index	0.804
Physical environment (9)	−0.22	0.09	0.77	4.47	0.346	Coefficient alpha	0.820
Financial support (12)	0.60	0.08	−1.97	10.48	0.033	Chi-square (Degrees of freedom)	36.97 (20)
Accessibility of information (13)	0.79	0.09	−1.09	5.33	0.255	*p-*value	0.0117
Leisure activity (14)	−0.28	0.08	1.23	4.46	0.347	Items fit residual (mean (SD))	−0.22 (1.32)
Home environment (23) ^a^						Persons fit residual (mean (SD))	−0.46 (1.06)
Health care (24) ^a^							
Transport (25) ^a^							

^a^Deleted items for e

**Fig. 1:**
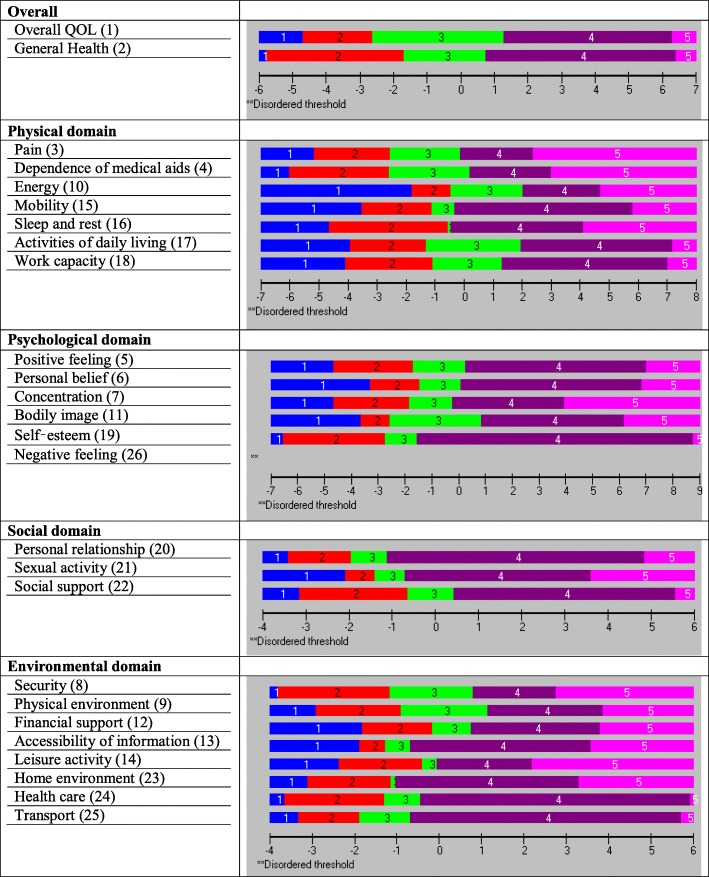
Initial threshold maps of the WHOQOL-BREF domains (sample size, *n* = 2425)

First two items (*overall QOL and general health)* of the WHOQOL-BREF scale showed misfit (Table 3). Preliminary analysis of 2425 participants for each of the four WHOQOL-BREF domains indicated that they did not meet the expectations of the Rasch model. Misfit was evidenced by a significant item-trait interaction for physical ((${\upchi}_{(28)}^2$ = 765.18, *p* < 0.001) and IFR (mean = − 1.09, SD = 6.12)), psychological ((${\upchi}_{(24)}^2$ = 843.59, *p* < 0.001) and IFR (mean = − 0.88, SD = 6.36)), social ((${\upchi}_{(12)}^2$ = 243.20, *p* < 0.001) and IFR (mean = − 1.65, SD = 2.63)) and environmental domain ((${\upchi}_{(32)}^2$ = 859.37, *p* < 0.001) and IFR (mean = − 0.35, SD = 6.25) (Table 3). Eighteen items were found misfit based on the overall fit as residual values were outside the range of ±2.5 and 24 items were found misfit based on the individual’s significant chi-square values (Table 3). However, only one item was disordered thresholds (Fig. 1) and ordered thresholds along could not implies the model fit. As discussed in the Methods section, for a very large sample size, a small deviation from the Rasch model results in a significantly large chi-square value, which influence model to be unfit, and the study manifested that evidence (Table 3). The Analysis was undertaken five times with five different random sub-samples, each of which was comprised of 300 participants and randomly selected from the core sample, to test the robustness of the scale. The five validation sub-samples exhibited similar results, so one illustrative sample was used for reporting purposes. Other sample results are provided in Additional files 1 and 2.

**Fig. 2:**
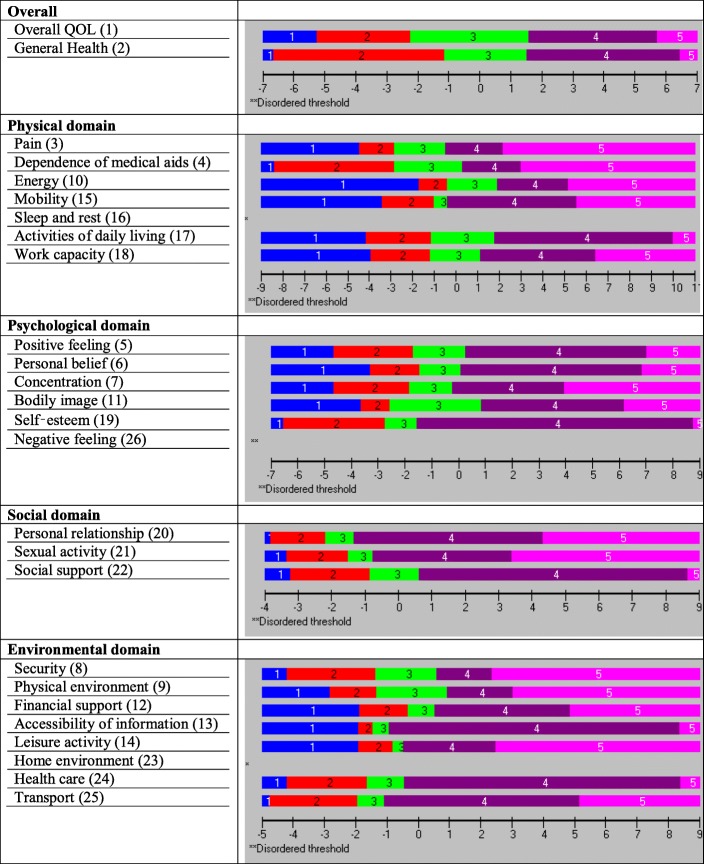
Initial threshold maps of the WHOQOL-BREF domains (sub-sample (for validation) size, *n* = 300)

The first two items on the WHOQOL-BREF (overall QOL and general health) appeared to be perfect fit (Table 4) and ordered thresholds (Fig. 2). Hence, these two items were not considered for any part of the domains and no further examination was performed. The following sub-sections discuss the results of the validation sample for four underlying domains.

### Physical domain subscale

**Fig. 3:**
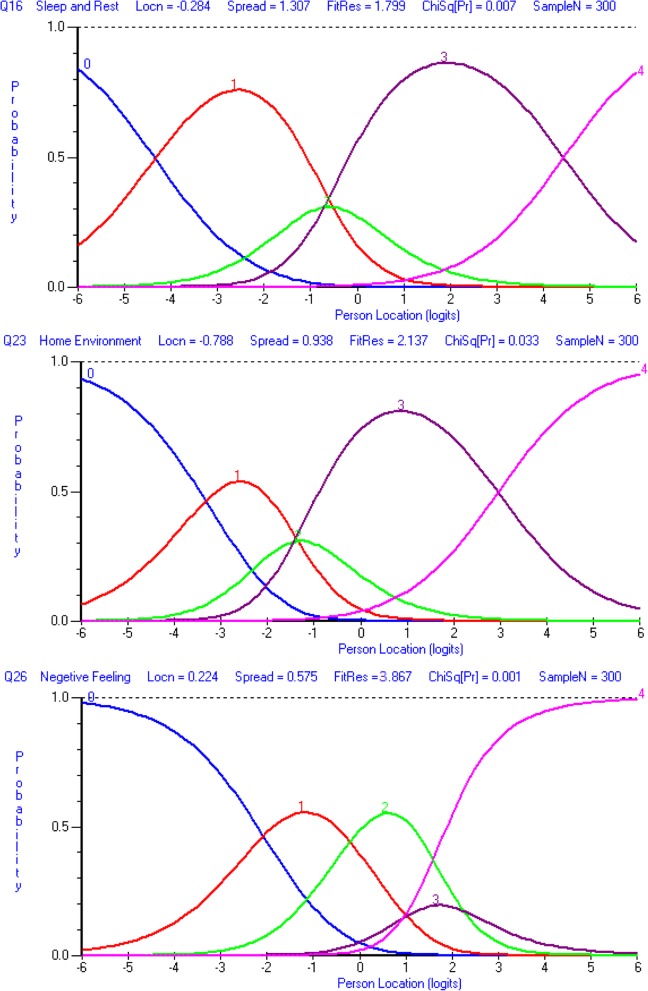
Category Probability Curve of the disorder thresholds items (sub-sample (for validation) size, *n* = 300)

**Fig. 4:**
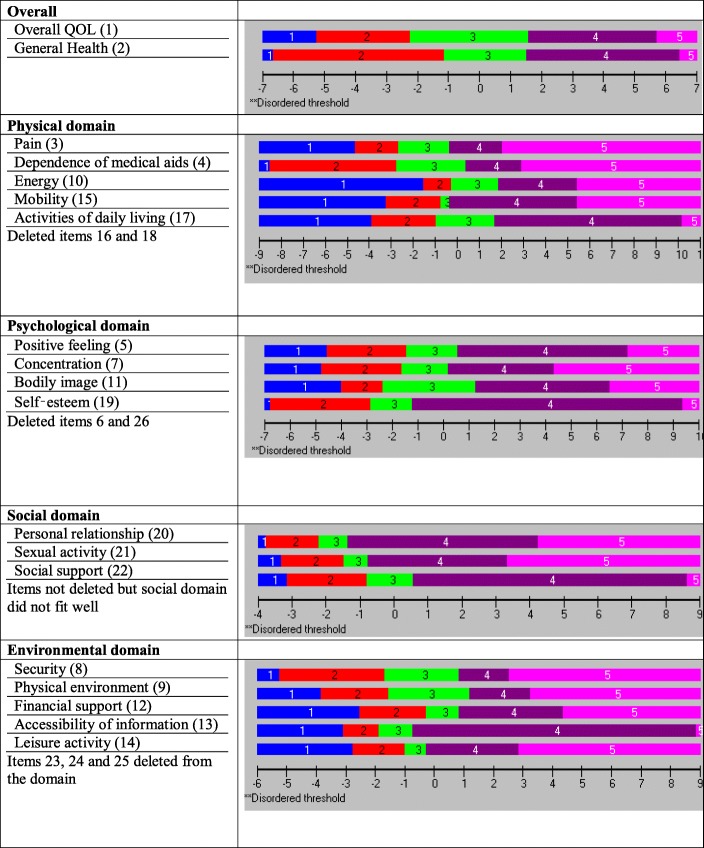
Final threshold maps of the modified WHOQOL-BREF domains (sub-sample (for validation) size, *n* = 300)

**Fig. 5:**
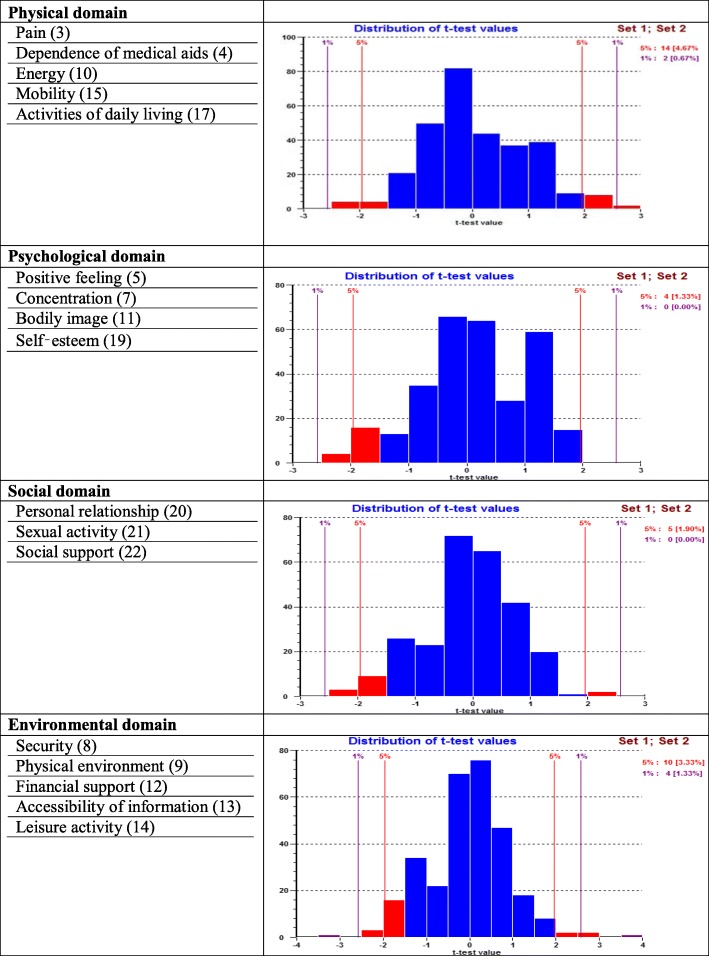
Dimensionality testing of the modified WHOQOL-BREF domains (sub-sample (for validation) size, *n* = 300)

The PSI for the original set of seven items was 0.834, indicating that the reliability of the physical domain was good (Table 4). All items showed ordered thresholds except for item 16 (*sleep and rest*) (Fig. 2); for the response options based on five-point Likert-type scales (0, 1, 2, 3, 4), category responses 2 and 3 (Fig. 3) did not have an equal distance across the trait. Initially, only the disordered item was rescored, and subsequently all items were rescored by merging the two middle categories; however, this did not improve the model fit, so the original scoring was retained. Overall, the model showed poor fit, as evident with the standardised item fit residuals (IFR) statistics (mean = − 0.34, SD = 2.39) and item-trait interaction statistics (${\upchi}_{(28)}^2$ = 107.18, *p* < 0.001) (Table 4). Using a Bonferroni adjustment, five items (item 3, 4, 15, 17 and 18) had significant chi-square *p-*values (Table 4). A few items were excluded as they exhibited highly significant chi-square *p-*values or high positive or negative residual values. Items were excluded stepwise one at a time during the overall fitness of the model and individual item statistics were checked after each iteration until a satisfactory model was achieved supported by a non-significant chi-square value. At first instance, item 16 (*sleep and rest*) was removed (high chi-square value and disordered thresholds). The deletion of the item 16 did not improve the overall model fit (${\upchi}_{(24)}^2$ = 78.68, *p* < 0.001) and individual item statistics were poor (mean = − 0.53, SD = 2.27). Next, the item 18 (*work capacity*) was excluded from the model due to high fit residuals value (− 4.20) as well as significant chi-square *p* value). However, it did not improve the overall model fit (${\upchi}_{(24)}^2$ = 73.92, p < 0.001). The deletion of these two items (16 and 18) resulted in an improved overall model fit with IFR (mean = − 0.30, SD = 1.12), Person Fit Residual (PFR) (mean = − 0.52, SD = 1.09) and total chi-square interaction statistics (${\upchi}_{(20)}^2$ = 36.47, *p* = 0.014) (Table 5). There was no evidence of DIF for the demographic variables (age and gender). The final PSI was 0.773 (CA = 0.790), suggesting sufficient person separation reliability for the revised five items in the physical domain; all individual item fit statistics were non-significant (Table 5) and all items had ordered thresholds (Fig. 4). The unidimensionality of the revised physical domain is supported by independent t-tests comparing the person estimates with the principal component analysis (PCA) of the residuals; our findings indicate that only 4.7% (95% Confidence Interval: 2.2 to 7.1%) of cases showed statistically significant differences (Fig. 5). The revised scale also had no local dependency, thus meeting the assumptions of the Rasch model.

### Psychological domain subscale

The PSI for the original set of six items was 0.767, indicating that the reliability of the psychological domain was good (Table 4). All items showed ordered thresholds except for item 26 (*negative feeling*) (Fig. 2); category response options 3 and 4 (Fig. 3) did not have an equal distance across the trait. Initially, one item was rescored at a time, and subsequently, all items were rescored by merging the two middle categories; however, this did not improve the model fit, so original scoring was retained. Overall, the model showed poor fit, as evident by IFR (mean = − 0.09, SD = 2.04) and item-trait interaction statistics (${\upchi}_{(24)}^2$ = 130.64, *p* < 0.001) (Table 4). Deleting item 6 (*personal belief*), due to a high chi-square value, did not improve the overall model fit (${\upchi}_{(20)}^2$ = 64.86, *p* < 0.001) and IFR (mean = − 0.53, SD = 1.93), suggesting more items needed to be removed. Further, deletion of item 26 (*negative feelings*) that occurred due to high fit residual value (3.87) as well as high chi-square value did not improve the model (${\upchi}_{(20)}^2$ = 56.72, *p* < 0.001). The deletion of both items 6 and 26 resulted in an improved overall model fit with IFR (mean = − 0.09, SD = 1.18), PFR (mean = − 0.30, SD = 0.86) and total chi-square interaction statistics (${\upchi}_{(16)}^2$ = 28.30, *p* = 0.029) (Table 5). There was no evidence of DIF for the demographic variables (age and gender). The final PSI was 0.708 (CA = 0.745), suggesting sufficient person separation reliability for the revised four item psychological domain; all individual item fit statistics were non-significant (Table 5) and all items had ordered thresholds (Fig. 4). The unidimensionality of the revised psychological domain was supported by independent t-tests comparing the person estimates with the PCA of the residuals and findings indicated that only 1.3% (95% Confidence Interval: 1.1 to 3.8%) of cases showed statistically significant differences (Fig. 5). There were no correlation coefficients above 0.30 on the person-item residual correlation matrix, indicating no local dependency of the items.

### Social domain subscale

The social domain met the other assumptions of the Rasch Model with no local dependency, ordered thresholds (Fig. 2), no DIF by age group or gender and no evidence of multidimensionality (Fig. 5). However, it had insufficient reliability PSI = 0.606 and which was evident from poor model fit (${\upchi}_{(12)}^2$ = 47.21, *p* < 0.001) (Table 4). No serious misfit was observed for both persons and items. Because of missing data, RUMM 2030 could not produce CA. Applying the Bonferroni adjustment to the *p-*values revealed that two items (20 and 22) were a misfit. Initially, 37 participants with missing data were removed from item 21 (*sexual activity*) and the domain was reanalysed using the remaining 263 participants. It improved the internal consistency and reliability (PSI =0.635 (CA =0.669) (Table 5) to represent a reasonable reliability fit. All other Rasch model assumptions remained same. As the domain had only three items, it was assessed whether reliability for Rasch assumptions could be deemed ‘reasonable’.

### Environment domain subscale

The environmental domain showed misfit to the model (${\upchi}_{(32)}^2$ = 173.69, *p* < 0.001, PSI = 0.740). Person fit statistics were within an acceptable range, but item fit statistics (mean = − 0.12, SD = 2.73) indicated the presence of misfitting items (Table 4). A disordered threshold was observed for item 23 (*home environment*) (Fig. 2); category response options 2 and 3 (Fig. 3) did not have an equal distance across the trait; however, rescoring did not result in improved model fit. Therefore, the original scoring was retained. Applying the Bonferroni adjustment to the *p-*values, four items (12, 13, 24 and 25) were found to have significant chi-square *p-*values, while item 25 had a high positive fit residual value (4.42), suggesting a misfit (Table 4). In order to improve the model fit, three items were deleted (23 (*home environment*) (due to high fit residuals value (2.14) and disordered thresholds), 24 (*health care*) (due to high chi-square value) and 25 (*transport*) (due to high fit residuals (4.42) and high chi-square values). Removing three items resulted in an improved overall model fit with IFR (mean = − 0.22, SD = 1.32), PFR (mean = − 0.46, SD = 1.06), and overall chi-square interaction statistics (${\upchi}_{(20)}^2$ = 36.97, *p* = 0.012). There was no evidence of DIF for the demographic variables (age and gender). The final PSI was 0.804 (CA = 0.820), suggesting sufficient person separation reliability for the revised five item environmental domain; all individual item fit statistics were non-significant(Table 5) and all items had ordered thresholds (Fig. 4). The distribution of the independent t-test value, comparing the person location for the two sets of items, indicated that only 3.33% of the test was significant. The associated binomial 95% Confidence Interval was 0.9 to 5.8. Thus, the presence of unidimensionality was supported (Fig. 5). This indicates that the scale measures only one construct. There were no correlation coefficients above 0.30 on the person-item residual correlation matrix, indicating no local dependency on the items.

### Targeting of the each of the four domains of the WHOQOL-BREF

**Fig. 6:**
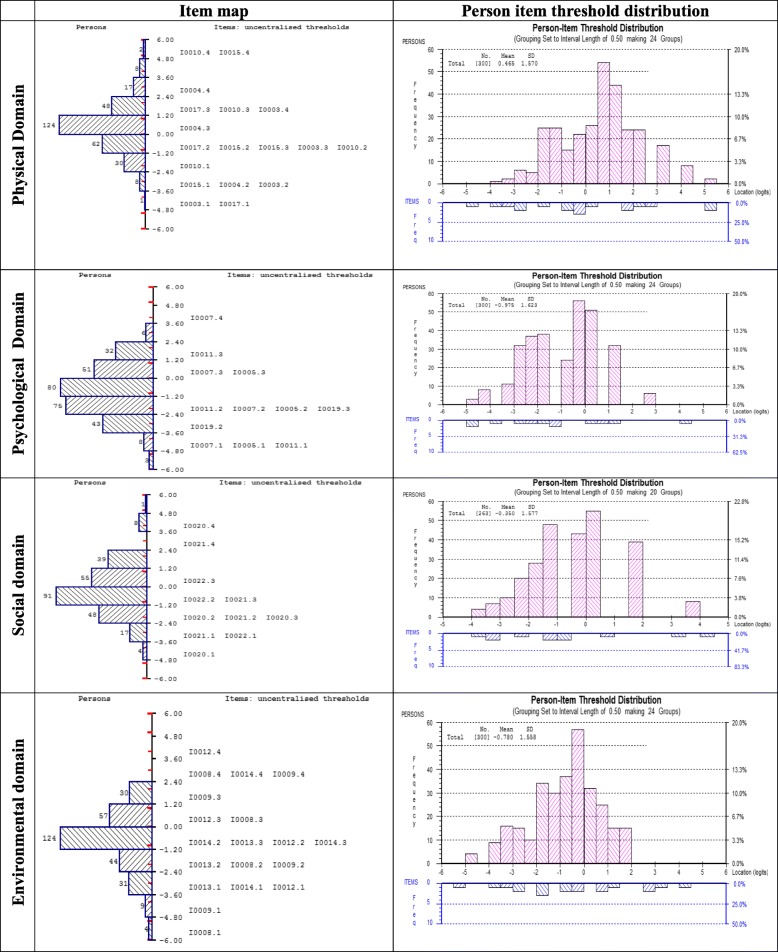
Item maps and person-item threshold distributions of the modified WHOQOL-BREF domains (sub-sample (for validation) size, *n* = 300)

Figure 6 presents the modified item map for the person-item threshold distribution of the four domains, showing targeting of the revised scale. The person distribution is shown in the top half and the item thresholds in the bottom half. The overall mean person logit for the physical domain was 0.466, the psychological domain was − 0.975, the social domain was − 0.350 and the environmental domain was − 0.780, suggesting well-targeted persons and items for each of the domains. On average, the physical domain showed a slightly higher level of quality of life, and the psychological, social and environmental domains showed slightly lower levels of quality of life than the average of the scale items. The person item distributions of the 5-item physical domain, 4-item psychological, 3-items of social and 5-item environmental domains are shown in Fig. 6. The modified versions of all four domains had better distributions of items across the range of quality of life scores than those observed with the original models.

## Discussion

Quality of life screening instruments are now widely used in both clinical practice and research. Increasingly, a mix of classical and modern psychometric approaches are used to scrutinise the measurement properties of scales. This investigation intended to apply a Rasch analysis to assess the psychometric properties of the WHOQOL-BREF scale, and specially, to evaluate the measurement properties of the four domains of the WHOQOL-BREF as a measure of QOL. To date, this could be deemed as the first study using the Rasch model to examine the psychometric properties of the WHOQOL-BREF in a large sample of adults, with a wide age distribution, from a typical rural district in Bangladesh. Key contributions made by this study include an assessment of the appropriateness of using all WHOQOL-BREF items to represent the underlying dimension of QOL, the fit of individual items and an assessment of the potential bias of subjects by gender and age. The utilisation of the Rasch measurement model in this study supported the viability of the revised version of the physical, psychological and environmental domains, but the social domain did not show a good fit in this population.

It is sensible to look for a contemporary scale that can fulfil assessment requirements rather than develop a new scale. Creating a new scale or checking existing processes using Rasch analysis are lengthy and rigorous processes. When all steps are completed, construction of a final model must undergo psychometric examination. A lot of time and exertion (and repeat testing) is required to develop a useful rating scale. However, the benefits of this undertaking extend beyond the individual project and support the entire clinical field. Modifying the WHOQOL-BREF through deletion of items to improve its performance using the Rasch model can r educe its comparability with previous WHOQOL-BREF studies. Nevertheless, this project was dedicated to maintaining the original structure as close as possible.

Initially, none of the four domains satisfied the criteria of fit for the Rasch model. To achieve a satisfactory fit, the study required to remove items. Items showing misfit were removed from each of the four domains gradually, after going through all possible steps to improve the model fit and considering each item’s performance in all phases of analysis. Item 16 (*sleep and rest*) and item 18 (*work capacity*) were removed from the physical domain. Removal of these two items from the domain resulted in adequate internal consistency, no evidence of multidimensionality, no DIF and no local dependency. The misfitting item in the physical domain (*sleep and rest*) is consistent with previous Rasch model findings where the reason they have been deleted was similar with our deletion criterion (large fit residuals and significant chi-square values) [69, 70].

Item 6 (*personal belief*) and item 26 (*negative feelings*) were removed from the psychological domain. Removal of these items from the model significantly improved the fit of the psychological domain and supported the design revision. The misfit of these items in the psychological domain contradicts previous Rasch model findings [69–72]. One possible reason for this is that respondents may have had difficulty understanding some items that used indirect wording (e.g., negatively worded items). Negatively worded items have a wording effect that biases the evaluation of the instrument [73]. Therefore, item 26 (*negative feelings*), a negatively worded item, may have affected the underlying constructs, which in turn affected the measurement of the psychological domain. Notably, negatively worded items did not affect the physical domain.

Item 25 (*transport*), item 24 (*health care)* and item 23 *(home environment)* were removed from the environmental domain gradually after going through all possible steps to improve the model fit. Removal of these items from the domain significantly improved the fit of the environment domain, therefore supporting this revision. Deletion of the *transport* and *home environment* items is consistent with a previous study using Rasch analysis where the reason they have deleted matched with our deletion criterion (large fit residuals and significant chi-square values) [69].

Several previous studies conducted in Bangladesh using CTT were unable to delete any items to achieve model fit [21–23]. For example, the Zeldenryk et al. study found 22 of the 26 questions problematic and identified limitations associated mainly with translation, wording and conceptual difficulties concluding that the WHOQOL-BREF was not suitable for use in rural Bangladesh. However, this study addressed some of the concerns raised and asserted that modified model could be applicable to rural Bangladesh. In summary, CTT studies such as the Zeldenryk et al. study did not explore the modus operandi of modifying the questionnaire for the proper fitness to use in the context of Bangladesh and consequently, comparison of this study with previous studies examining problematic items in the WHOQOL-BREF is, by necessity, somewhat limited.

Removal of items from the scale eliminates at least some redundancy or vice versa [51, 74–76]. While removal of items from the physical and psychological domains reduced the CA and PSI, it increased the CA and PSI for the environmental domain. For the physical domain, the 5-item scale CA (0.79) and PSI (0.77) were close to the original 7-item scale CA (0.85) and PSI (0.83). For the psychological domain, the revised 4-item scale CA (0.74) and PSI (0.70) were close to the original 6-item scale CA (0.77) and PSI (0.76). For the environment domain, the 5-item scale CA (0.82) and PSI (0.80) exceeded the original 8-item scale CA (0.73) and PSI (0.74). To improve the model fit in the social domain, missing responses were deleted, and the scale was reanalysed. However, while the CA and PSI improved, the scale still did not fulfil the Rasch property of reliability. Moreover, several studies raised significant concerns about social domain in both developed and developing countries. [69, 77]. However, in case of social domain, PSI appeared to be much better than other studies [69, 72, 77]. The proposed model for this research for the environment domain showed considerably greater reliability compared with the original domain, confirming adequate fit in the rural setting in Bangladesh.

This study has provided the first reliable data validating the WHOQOL-BREF- scale among the general population within rural districts in Bangladesh. This study was conducted among a large sample of adults and older adults across wide ranging ages. Data was collected directly by face-to-face interviews. This research project illustrates how the Rasch model can be utilised for thorough examination and improvement of measurement instruments within the WHOQOL-BREF scale. The Rasch analysis improves estimation accuracy, for example, lack of invariance, which has been overlooked in traditional analyses [51, 78]. The Rasch analysis of the WHOQOL-BREF scale indicates that the psychometric properties of the original scale among the population in Bangladesh would most likely have been much better if the scale development had been guided by IRT (Rasch analyses). Decreasing the number of items may improve the properties of the scale and may make it easier for participants to give more truthful answers [51, 79].

The findings of this study raise significant questions about the appropriateness of two of the three items of the WHOQOL-BREF social domain scale for older (especially widowed) and younger (especially unmarried) participants. The social domain contains only three items and one of these items relates to sex life, which may not be relevant to older, widowed and unmarried people. From a cultural and social point of view, in South-East Asia, many people believe that without marriage, sex is a social crime. Even though some respondents in these age categories may be engaged, or have an excellent sex life without marriage, they feel shy and reluctant to give appropriate answers. In 2003, Gott et al. [80] raised this issue, but no alternative has been proposed in the literature so far as it seems quite challenging to identify solutions for such established customs in developing countries and religious-based nations like Indonesia, Bangladesh and Middle-eastern countries. In addition, the respondents did not properly understand the item associated with “personal relationships”; thus, the real meaning of the responses was subsequently challenging to determine. The application of this result is partially limited by the fact that the study was performed in a single rural area of a developing country. The scale was designed to be applicable cross-culturally and further work is needed in similar culture of developing countries. Reliability was low due to short length of this subscale as well as missing data. Further research is needed to test the integrity of such responses and may be overcome by using an alternative method for sensitive question (item 21 (*Sex life*)).

The present study did not re-examine the original 4-domain structure of the WHOQOL-BREF, and this may be a limitation. However, most of the validated cross-cultural studies have found that the four-factor structure of the WHOQOL-BREF is the most appropriate structure [69]. The response rate for each of the 26 items of the WHOQOL-BREF was 100%, except for item 21 (91%) (*sex life*). The acceptable proportion of missing data was reported to be < 5% [81] to < 10% [19]; thus, our missing data rate was within an acceptable range. Moreover, the RUMM 2030 program can effectively mitigate this limitation as it can handle missing item response data [54]. Omission of the ‘work capacity’ poses significant limitation to the shaping of physical domain as it linked with level of education and socio-economic condition of study population, which merits further research. Moreover, the perception of home environment, access to health care and transportation pertaining to the environment domain could be better addressed in a future study by using modified questionnaire to gauge the impact of literacy on perceptions of home, access to health care and transportation. A potential drawback of these findings based on a single-occasion collection of data from a rural district in Bangladesh is that the findings may not be truly reflective of the national rural perspective due to different demographic and cultural characteristics across the country. However, it should be noted that the socio-demographic characteristics of the Narail district are similar to those in most of the rural areas in Bangladesh.

## Conclusion

In conclusion, this research verifies that the WHOQOL-BREF is somewhat inadequate for measuring QOL among adults in rural Bangladesh. However, the revised scale (physical, psychological and environmental domains) showed promising internal validity when evaluated under the rigorous assumptions of the Rasch measurement model. Although, the social domain did not reach the strict assumption of reliability within Rasch analysis. However, it shows a reasonable fit without missing data. This opens an avenue for further research to redevelop the social domain. Application of Rasch modelling is only an initial step. Further validation studies of this revised scale against a clinical assessment analysis of the modified questionnaire structure are needed (concerning its acceptability, validity, reliability and responsiveness). The findings of this investigation are preliminary. Further research should include a revised version of the WHOQOL-BREF scale and give specific attention to the social domain, particularly with respect to appropriate sample selection for item 21 (*sex life*) and the methods of interview process.

## Additional files

Additional file 1: Performance of the Rasch analysis of the WHOQOL-BREF domains (other four sub-samples of size *n* = 300 each) (DOCX 36 kb)

Additional file 2: Threshold maps of the WHOQOL-BREF domains (other four sub-samples of size *n* = 300 each) (DOCX 226 kb)

## Abbreviations

CA: Cronbach’s Alpha
CTT: Classical Test Theory
DIF: Differential Item Functioning
IFR: Item Fit Residuals
PCA: Principal Component Analysis
PFR: Person Fit Residuals
PSI: Person Separation Index
QOL: Quality of Life
WHOQOL-BREF: The World Health Organisation Quality of Life Instruments, Short Form
