# Supplementary material for: Psychometric evaluation of an interview-administered version of the WHOQOL-BREF questionnaire for use in a cross-sectional study of a rural district in Bangladesh: an application of Rasch analysis
Source: BMC Health Serv Res. 2019 Apr 5;19:216. doi: 10.1186/s12913-019-4026-0 (PMC6451264; doi:10.1186/s12913-019-4026-0)
Supplement: Supplementary file 1 — Performance of the Rasch analysis of the WHOQOL-BREF domains (other four sub-samples of size n = 300 each) (DOCX 36 kb) [file 12913_2019_4026_MOESM1_ESM.docx]

**Additional file 1**

**Table A: Performance of the Rasch analysis of the WHOQOL-BREF domains (first sub-sample (for validation) size, n = 300)**

| **WHOQOL-BREF scales** | **Original instrument** | | | | | **Summary of overall model fit statistics of each domain**  **(original instrument)** | |
| --- | --- | --- | --- | --- | --- | --- | --- |
|  | **Location** | **S.E.** | **Residual** | **χ^2^** | ***p*-value** |  |  |
| **Overall** |  |  |  |  |  |  |  |
| **Overall QOL** | -0.03 | 0.10 | -1.21 | 3.25 | 0.516 |  |  |
| **General Health** | 0.02 | 0.11 | -1.32 | 4.59 | 0.332 |  |  |
| **Physical domain** |  |  |  |  |  |  |  |
| Pain (3) | -2.32 | 0.10 | -1.85 | 10.30 | 0.036 | Person separation index | 0.892 |
| Dependence on medical aids (4) | -2.32 | 0.11 | 1.76 | 24.23 | 0.000 | Coefficient alpha | 0.915 |
| Energy (10) | 0.79 | 0.11 | 1.20 | 23.83 | 0.000 | Chi-square (Degrees of freedom) | 113.34 (28) |
| Mobility (15) | 1.47 | 0.11 | -0.04 | 12.64 | 0.013 | *p-*value | 0.000000 |
| Sleep and rest (16) | -0.34 | 0.11 | 0.67 | 14.24 | 0.007 | Items fit residual (mean (SD)) | -0.79 (2.35) |
| Activities of daily living (17) | 1.51 | 0.12 | -4.81 | 17.77 | 0.001 | Persons fit residual (mean (SD)) | -0.55 (1.16) |
| Work capacity (18) | 1.21 | 0.12 | -2.45 | 10.35 | 0.035 |  |  |
| **Psychological domain** |  |  |  |  |  |  |  |
| Positive feeling (5) | 0.22 | 0.11 | -1.43 | 14.36 | 0.006 | Person separation index | 0.706 |
| Personal belief (6) | 0.23 | 0.09 | 1.54 | 83.76 | 0.000 | Coefficient alpha | 0.714 |
| Concentration (7) | 0.23 | 0.10 | -2.41 | 24.36 | 0.000 | Chi-square (Degrees of freedom) | 187.76 (24) |
| Bodily image (11) | -1.30 | 0.11 | 0.41 | 16.88 | 0.002 | *p-*value | 0.000000 |
| Self-esteem (19) | -1.49 | 0.10 | 1.53 | 33.99 | 0.000 | Items fit residual (mean (SD)) | 0.16 (1.69) |
| Negative feeling (26) | 2.11 | 0.10 | 1.33 | 14.43 | 0.006 | Persons fit residual (mean (SD)) | -0.20 (0.88) |
| **Social domain** |  |  |  |  |  |  |  |
| Personal relationship (20) | -0.67 | 0.10 | -1.55 | 21.44 | 0.000 | Person separation index | 0.533 |
| Sexual activity (21) | -0.25 | 0.08 | -1.40 | 7.95 | 0.094 | Coefficient alpha | * |
| Social support (22) | 0.92 | 0.10 | 0.40 | 20.04 | 0.000 | Chi-square (Degrees of freedom) | 49.42 (12) |
|  |  |  |  |  |  | *p-*value | 0.000002 |
|  |  |  |  |  |  | Items fit residual (mean (SD)) | -0.85 (1.09) |
|  |  |  |  |  |  | Persons fit residual (mean (SD)) | -0.29 (0.54) |
| **Environmental domain** |  |  |  |  |  |  |  |
| Security (8) | 0.46 | 0.10 | -1.37 | 15.79 | 0.003 | Person separation index | 0.747 |
| Physical environment (9) | 0.20 | 0.10 | 0.53 | 14.55 | 0.006 | Coefficient alpha | 0.702 |
| Financial support (12) | 0.39 | 0.08 | -3.13 | 23.99 | 0.000 | Chi-square (Degrees of freedom) | 162.19 (32) |
| Accessibility of information (13) | -0.18 | 0.09 | -1.98 | 21.34 | 0.000 | *p-*value | 0.000000 |
| Leisure activity (14) | -0.31 | 0.08 | 0.34 | 6.74 | 0.150 | Items fit residual (mean (SD)) | 0.01 (2.04) |
| Home environment (23) | -0.41 | 0.07 | 3.12 | 51.95 | 0.000 | Persons fit residual (mean (SD)) | -0.23 (0.98) |
| Health care (24) | 0.02 | 0.09 | 1.09 | 12.71 | 0.013 |  |  |
| Transport (25) | -0.16 | 0.11 | 1.48 | 15.14 | 0.004 |  |  |

## *Coefficient of Alpha cannot be calculated on missing data

**Table B: Performance of the Rasch analysis of the WHOQOL-BREF domains (third sub-sample (for validation) size, n = 300)**

| **WHOQOL-BREF scales** | **Original instrument** | | | | | **Summary of overall model fit statistics of each domain**  **(original instrument)** | |
| --- | --- | --- | --- | --- | --- | --- | --- |
|  | **Location** | **S.E.** | **Residual** | **χ^2^** | ***p*-value** |  |  |
| **Overall** |  |  |  |  |  |  |  |
| **Overall QOL** | -0.05 | 0.15 | -1.26 | 3.90 | 0.419 |  |  |
| **General Health** | 0.06 | 0.17 | -1.22 | 4.01 | 0.404 |  |  |
| **Physical domain** |  |  |  |  |  |  |  |
| Pain (3) | -2.35 | 0.10 | 0.08 | 16.32 | 0.003 | Person separation index | 0.893 |
| Dependence on medical aids (4) | -2.36 | 0.11 | 2.39 | 43.34 | 0.000 | Coefficient alpha | 0.916 |
| Energy (10) | 1.22 | 0.11 | 0.97 | 14.80 | 0.005 | Chi-square (Degrees of freedom) | 141.07 (25) |
| Mobility (15) | 0.44 | 0.10 | -1.21 | 16.25 | 0.003 | *p-*value | 0.000000 |
| Sleep and rest (16) | -0.06 | 0.10 | -2.58 | 17.39 | 0.002 | Items fit residual (mean (SD)) | -1.04 (2.47) |
| Activities of daily living (17) | 1.68 | 0.12 | -5.09 | 24.72 | 0.000 | Persons fit residual (mean (SD)) | -0.65 (1.23) |
| Work capacity (18) | 1.42 | 0.12 | -1.87 | 8.25 | 0.083 |  |  |
| **Psychological domain** |  |  |  |  |  |  |  |
| Positive feeling (5) | 0.42 | 0.12 | -1.97 | 13.77 | 0.008 | Person separation index | 0.708 |
| Personal belief (6) | 0.98 | 0.09 | 2.82 | 69.64 | 0.000 | Coefficient alpha | 0.718 |
| Concentration (7) | -0.68 | 0.11 | -2.91 | 24.53 | 0.000 | Chi-square (Degrees of freedom) | 184.78 (24) |
| Bodily image (11) | 0.00 | 0.11 | 0.07 | 9.70 | 0.046 | *p-*value | 0.000000 |
| Self-esteem (19) | -1.42 | 0.10 | 2.06 | 54.06 | 0.000 | Items fit residual (mean (SD)) | 0.25 (2.29) |
| Negative feeling (26) | 0.71 | 0.09 | 1.42 | 13.09 | 0.011 | Persons fit residual (mean (SD)) | -0.26 (0.88) |
| **Social domain** |  |  |  |  |  |  |  |
| Personal relationship (20) | 0.59 | 0.09 | -1.56 | 18.83 | 0.001 | Person separation index | 0.404 |
| Sexual activity (21) | -0.27 | 0.08 | -0.59 | 8.61 | 0.072 | Coefficient alpha | * |
| Social support (22) | -0.33 | 0.09 | 0.69 | 24.05 | 0.000 | Chi-square (Degrees of freedom) | 51.49 (12) |
|  |  |  |  |  |  | *p-*value | 0.000001 |
|  |  |  |  |  |  | Items fit residual (mean (SD)) | -0.49 (1.13) |
|  |  |  |  |  |  | Persons fit residual (mean (SD)) | -0.32 (0.61) |
| **Environmental domain** |  |  |  |  |  |  |  |
| Security (8) | -0.11 | 0.10 | -1.28 | 16.16 | 0.003 | Person separation index | 0.727 |
| Physical environment (9) | 0.12 | 0.10 | -0.03 | 14.35 | 0.006 | Coefficient alpha | 0.690 |
| Financial support (12) | 0.41 | 0.08 | -2.69 | 16.85 | 0.002 | Chi-square (Degrees of freedom) | 149.06 (32) |
| Accessibility of information (13) | -0.31 | 0.09 | -1.82 | 17.92 | 0.001 | *p-*value | 0.000000 |
| Leisure activity (14) | -0.40 | 0.07 | -0.18 | 8.20 | 0.085 | Items fit residual (mean (SD)) | 0.07 (2.05) |
| Home environment (23) | -0.45 | 0.07 | 3.57 | 53.28 | 0.000 | Persons fit residual (mean (SD)) | -0.27 (1.05) |
| Health care (24) | -0.17 | 0.10 | 1.64 | 13.84 | 0.008 |  |  |
| Transport (25) | 0.92 | 0.10 | 1.32 | 8.45 | 0.076 |  |  |

## *Coefficient of Alpha cannot be calculated on missing data

**Table C: Performance of the Rasch analysis of the WHOQOL-BREF domains (fourth sub-sample (for validation) size, n = 300)**

| **WHOQOL-BREF scales** | **Original instrument** | | | | | **Summary of overall model fit statistics of each domain**  **(original instrument)** | |
| --- | --- | --- | --- | --- | --- | --- | --- |
|  | **Location** | **S.E.** | **Residual** | **χ^2^** | ***p*-value** |  |  |
| **Overall** |  |  |  |  |  |  |  |
| **Overall QOL** | -0.09 | 0.10 | -1.39 | 2.10 | 0.717 |  |  |
| **General Health** | 0.08 | 0.14 | -1.33 | 5.79 | 0.215 |  |  |
| **Physical domain** |  |  |  |  |  |  |  |
| Pain (3) | -1.12 | 0.09 | -0.11 | 11.19 | 0.025 | Person separation index | 0.832 |
| Dependence on medical aids (4) | -1.87 | 0.10 | 1.55 | 10.36 | 0.035 | Coefficient alpha | 0.849 |
| Energy (10) | 1.33 | 0.09 | 2.77 | 13.82 | 0.008 | Chi-square (Degrees of freedom) | 102.21 (28) |
| Mobility (15) | 0.23 | 0.10 | -1.43 | 11.89 | 0.018 | *p-*value | 0.000000 |
| Sleep and rest (16) | -0.41 | 0.09 | 1.64 | 22.36 | 0.000 | Items fit residual (mean (SD)) | 0.15 (1.92) |
| Activities of daily living (17) | 1.02 | 0.11 | -2.59 | 24.71 | 0.000 | Persons fit residual (mean (SD)) | -0.45 (1.19) |
| Work capacity (18) | 0.82 | 0.10 | -0.82 | 7.89 | 0.096 |  |  |
| **Psychological domain** |  |  |  |  |  |  |  |
| Positive feeling (5) | 0.42 | 0.10 | -2.32 | 29.43 | 0.000 | Person separation index | 0.727 |
| Personal belief (6) | 0.71 | 0.09 | -1.78 | 25.85 | 0.000 | Coefficient alpha | 0.740 |
| Concentration (7) | -0.78 | 0.09 | -1.41 | 13.36 | 0.010 | Chi-square (Degrees of freedom) | 129.77 (24) |
| Bodily image (11) | 0.35 | 0.09 | 1.24 | 18.75 | 0.001 | *p-*value | 0.000000 |
| Self-esteem (19) | -0.93 | 0.09 | 1.44 | 25.31 | 0.000 | Items fit residual (mean (SD)) | -0.19 (1.83) |
| Negative feeling (26) | 0.23 | 0.08 | 1.69 | 17.07 | 0.002 | Persons fit residual (mean (SD)) | -0.29 (0.94) |
| **Social domain** |  |  |  |  |  |  |  |
| Personal relationship (20) | -0.21 | 0.09 | -1.86 | 25.46 | 0.000 | Person separation index | 0.545 |
| Sexual activity (21) | -0.20 | 0.08 | -0.42 | 3.93 | 0.416 | Coefficient alpha | * |
| Social support (22) | 0.40 | 0.08 | 0.37 | 18.68 | 0.001 | Chi-square (Degrees of freedom) | 48.07 (12) |
|  |  |  |  |  |  | *p-*value | 0.000003 |
|  |  |  |  |  |  | Items fit residual (mean (SD)) | -0.63 (1.13) |
|  |  |  |  |  |  | Persons fit residual (mean (SD)) | -0.33 (0.62) |
| **Environmental domain** |  |  |  |  |  |  |  |
| Security (8) | -0.40 | 0.08 | -0.46 | 12.12 | 0.016 | Person separation index | 0.682 |
| Physical environment (9) | 0.00 | 0.09 | -0.36 | 4.33 | 0.363 | Coefficient alpha | 0.678 |
| Financial support (12) | 0.42 | 0.07 | -2.96 | 25.51 | 0.000 | Chi-square (Degrees of freedom) | 125.08 (32) |
| Accessibility of information (13) | -0.10 | 0.07 | -2.27 | 22.46 | 0.000 | *p-*value | 0.000000 |
| Leisure activity (14) | -0.25 | 0.07 | -0.04 | 4.97 | 0.291 | Items fit residual (mean (SD)) | 0.03 (2.01) |
| Home environment (23) | -0.36 | 0.08 | 1.54 | 7.21 | 0.125 | Persons fit residual (mean (SD)) | -0.23 (1.02) |
| Health care (24) | 0.94 | 0.08 | 2.15 | 21.69 | 0.000 |  |  |
| Transport (25) | -0.25 | 0.09 | 2.64 | 26.80 | 0.000 |  |  |

## *Coefficient of Alpha cannot be calculated on missing data

**Table D: Performance of the Rasch analysis of the WHOQOL-BREF domains (fifth sub-sample (for validation) size, n = 300)**

| **WHOQOL-BREF scales** | **Original instrument** | | | | | **Summary of overall model fit statistics of each domain**  **(original instrument)** | |
| --- | --- | --- | --- | --- | --- | --- | --- |
|  | **Location** | **S.E.** | **Residual** | **χ^2^** | ***p*-value** |  |  |
| **Overall** |  |  |  |  |  |  |  |
| **Overall QOL** | -0.03 | 0.09 | -1.40 | 5.50 | 0.239 |  |  |
| **General Health** | 0.07 | 0.18 | -1.39 | 3.41 | 0.491 |  |  |
| **Physical domain** |  |  |  |  |  |  |  |
| Pain (3) | -2.13 | 0.09 | -0.82 | 13.80 | 0.008 | Person separation index | 0.824 |
| Dependence on medical aids (4) | -2.07 | 0.10 | 1.81 | 7.85 | 0.097 | Coefficient alpha | 0.843 |
| Energy (10) | 0.81 | 0.09 | 2.56 | 19.58 | 0.001 | Chi-square (Degrees of freedom) | 103.25 (28) |
| Mobility (15) | 0.51 | 0.10 | -1.18 | 6.85 | 0.144 | *p-*value | 0.000000 |
| Sleep and rest (16) | -0.36 | 0.09 | 1.36 | 25.62 | 0.000 | Items fit residual (mean (SD)) | -0.14 (2.05) |
| Activities of daily living (17) | 1.73 | 0.11 | -2.87 | 19.04 | 0.001 | Persons fit residual (mean (SD)) | -0.45 (1.15) |
| Work capacity (18) | 1.51 | 0.10 | -1.85 | 10.52 | 0.033 |  |  |
| **Psychological domain** |  |  |  |  |  |  |  |
| Positive feeling (5) | 0.36 | 0.10 | -2.42 | 27.13 | 0.000 | Person separation index | 0.746 |
| Personal belief (6) | 0.69 | 0.09 | -1.10 | 29.93 | 0.000 | Coefficient alpha | 0.760 |
| Concentration (7) | 0.33 | 0.09 | -2.25 | 17.90 | 0.001 | Chi-square (Degrees of freedom) | 119.66 (24) |
| Bodily image (11) | -0.88 | 0.10 | 1.28 | 20.76 | 0.000 | *p-*value | 0.000000 |
| Self-esteem (19) | -1.35 | 0.10 | 1.68 | 11.74 | 0.019 | Items fit residual (mean (SD)) | 0.00 (2.21) |
| Negative feeling (26) | 0.85 | 0.09 | 2.81 | 12.20 | 0.016 | Persons fit residual (mean (SD)) | -0.26 (0.99) |
| **Social domain** |  |  |  |  |  |  |  |
| Personal relationship (20) | -0.53 | 0.09 | -1.66 | 23.12 | 0.000 | Person separation index | 0.497 |
| Sexual activity (21) | -0.10 | 0.07 | -0.98 | 7.91 | 0.095 | Coefficient alpha | * |
| Social support (22) | 0.63 | 0.08 | 0.39 | 16.35 | 0.003 | Chi-square (Degrees of freedom) | 47.38 (12) |
|  |  |  |  |  |  | *p-*value | 0.000004 |
|  |  |  |  |  |  | Items fit residual (mean (SD)) | -0.75 (1.05) |
|  |  |  |  |  |  | Persons fit residual (mean (SD)) | -0.34 (0.68) |
| **Environmental domain** |  |  |  |  |  |  |  |
| Security (8) | -0.71 | 0.09 | -0.46 | 9.59 | 0.048 | Person separation index | 0.722 |
| Physical environment (9) | -0.05 | 0.09 | 0.30 | 3.09 | 0.543 | Coefficient alpha | 0.715 |
| Financial support (12) | 0.49 | 0.08 | -3.38 | 31.97 | 0.000 | Chi-square (Degrees of freedom) | 104.89 (32) |
| Accessibility of information (13) | -0.21 | 0.07 | -2.12 | 9.69 | 0.046 | *p-*value | 0.000000 |
| Leisure activity (14) | -0.17 | 0.07 | -0.06 | 1.73 | 0.785 | Items fit residual (mean (SD)) | 0.02 (2.06) |
| Home environment (23) | -0.61 | 0.08 | 1.33 | 14.93 | 0.005 | Persons fit residual (mean (SD)) | -0.20 (0.94) |
| Health care (24) | 0.31 | 0.08 | 1.46 | 7.55 | 0.110 |  |  |
| Transport (25) | 0.94 | 0.09 | 3.10 | 26.35 | 0.000 |  |  |

## *Coefficient of Alpha cannot be calculated on missing data
