# Supplementary material for: Psychometric evaluation of an interview-administered version of the WHOQOL-BREF questionnaire for use in a cross-sectional study of a rural district in Bangladesh: an application of Rasch analysis
Source: BMC Health Serv Res. 2019 Apr 5;19:216. doi: 10.1186/s12913-019-4026-0 (PMC6451264; doi:10.1186/s12913-019-4026-0)
Supplement: Supplementary file 2 — Threshold maps of the WHOQOL-BREF domains (other four sub-samples of size n = 300 each) (DOCX 226 kb) [file 12913_2019_4026_MOESM2_ESM.docx]

**Additional file 2**

**Figure A: Threshold maps of the WHOQOL-BREF domains (first sub-sample (for validation) size, n = 300)**

| **Physical domain** |  |
| --- | --- |
| \| Pain (3) \| \| --- \| \| Dependence of medical aids (4) \| \| Energy (10) \| \| Mobility (15) \| \| Sleep and rest (16) \| \| Activities of daily living (17) \| \| Work capacity (18) \| | 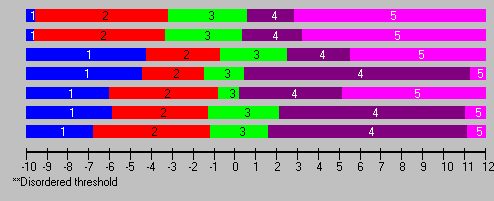 |
| **Psychological domain** |  |
| \| Positive feeling (5) \| \| --- \| \| Personal belief (6) \| \| Concentration (7) \| \| Bodily image (11) \| \| Self‑esteem (19) \| \| Negative feeling (26) \| | 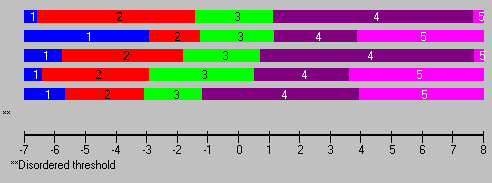 |
| **Social domain** |  |
| \| Personal relationship (20) \| \| --- \| \| Sexual activity (21) \| \| Social support (22) \| | 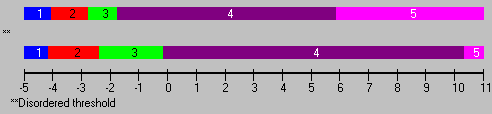 |
| **Environmental domain** |  |
| \| Security (8) \| \| --- \| \| Physical environment (9) \| \| Financial support (12) \| \| Accessibility of information (13) \| \| Leisure activity (14) \| \| Home environment (23) \| \| Health care (24) \| \| Transport (25) \| | 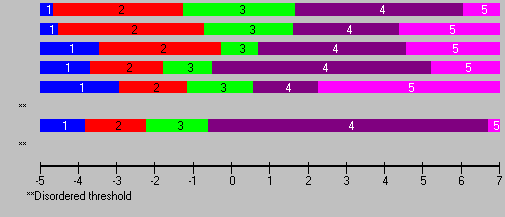 |

**Figure B: Threshold maps of the WHOQOL-BREF domains (third sub-sample (for validation) size, n = 300)**

| **Physical domain** |  |
| --- | --- |
| \| Pain (3) \| \| --- \| \| Dependence of medical aids (4) \| \| Energy (10) \| \| Mobility (15) \| \| Sleep and rest (16) \| \| Activities of daily living (17) \| \| Work capacity (18) \| | 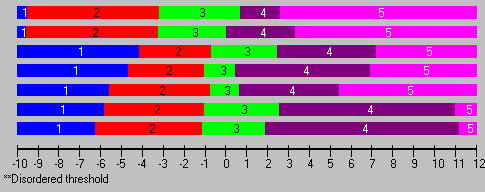 |
| **Psychological domain** |  |
| \| Positive feeling (5) \| \| --- \| \| Personal belief (6) \| \| Concentration (7) \| \| Bodily image (11) \| \| Self‑esteem (19) \| \| Negative feeling (26) \| | 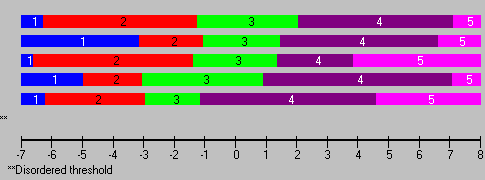 |
| **Social domain** |  |
| \| Personal relationship (20) \| \| --- \| \| Sexual activity (21) \| \| Social support (22) \| | 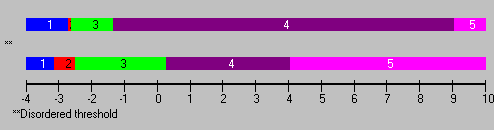 |
| **Environmental domain** |  |
| \| Security (8) \| \| --- \| \| Physical environment (9) \| \| Financial support (12) \| \| Accessibility of information (13) \| \| Leisure activity (14) \| \| Home environment (23) \| \| Health care (24) \| \| Transport (25) \| | 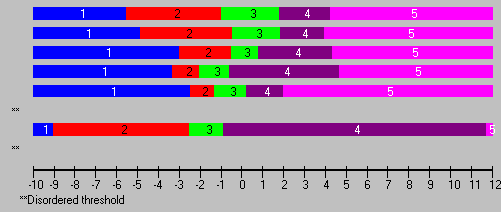 |

**Figure C: Threshold maps of the WHOQOL-BREF domains (fourth sub-sample (for validation) size, n = 300)**

| **Physical domain** |  |
| --- | --- |
| \| Pain (3) \| \| --- \| \| Dependence of medical aids (4) \| \| Energy (10) \| \| Mobility (15) \| \| Sleep and rest (16) \| \| Activities of daily living (17) \| \| Work capacity (18) \| | 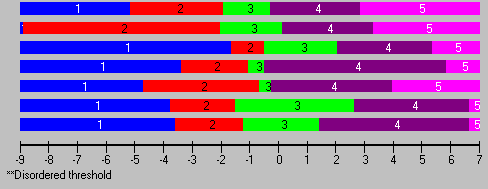 |
| **Psychological domain** |  |
| \| Positive feeling (5) \| \| --- \| \| Personal belief (6) \| \| Concentration (7) \| \| Bodily image (11) \| \| Self‑esteem (19) \| \| Negative feeling (26) \| | 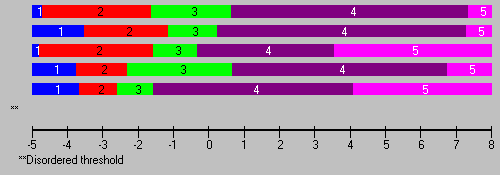 |
| **Social domain** |  |
| \| Personal relationship (20) \| \| --- \| \| Sexual activity (21) \| \| Social support (22) \| | 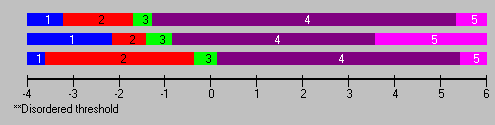 |
| **Environmental domain** |  |
| \| Security (8) \| \| --- \| \| Physical environment (9) \| \| Financial support (12) \| \| Accessibility of information (13) \| \| Leisure activity (14) \| \| Home environment (23) \| \| Health care (24) \| \| Transport (25) \| | 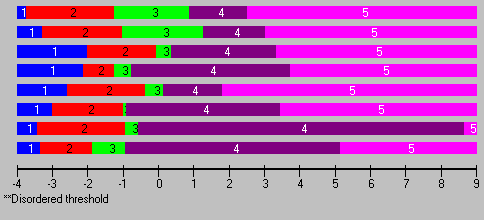 |

**Figure D: Threshold maps of the WHOQOL-BREF domains (fifth sub-sample (for validation) size, n = 300)**

| **Physical domain** |  |
| --- | --- |
| \| Pain (3) \| \| --- \| \| Dependence of medical aids (4) \| \| Energy (10) \| \| Mobility (15) \| \| Sleep and rest (16) \| \| Activities of daily living (17) \| \| Work capacity (18) \| | 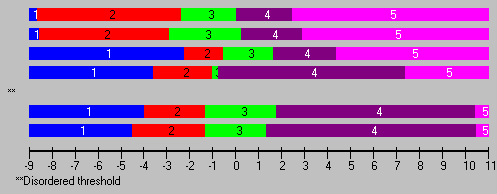 |
| **Psychological domain** |  |
| \| Positive feeling (5) \| \| --- \| \| Personal belief (6) \| \| Concentration (7) \| \| Bodily image (11) \| \| Self‑esteem (19) \| \| Negative feeling (26) \| | 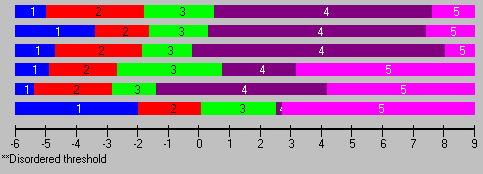 |
| **Social domain** |  |
| \| Personal relationship (20) \| \| --- \| \| Sexual activity (21) \| \| Social support (22) \| | 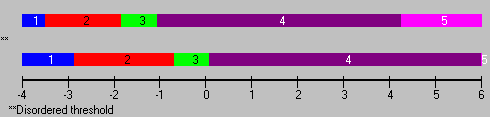 |
| **Environmental domain** |  |
| \| Security (8) \| \| --- \| \| Physical environment (9) \| \| Financial support (12) \| \| Accessibility of information (13) \| \| Leisure activity (14) \| \| Home environment (23) \| \| Health care (24) \| \| Transport (25) \| | 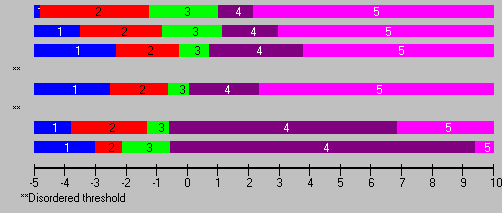 |
